# Collaborative Damage Detection Framework for Rail Structures Based on a Multi-Agent System Embedded with Soft Multi-Functional Sensors

**DOI:** 10.3390/s22207795

**Published:** 2022-10-14

**Authors:** Xiao Cheng, Daojin Yao, Lin Yang, Wentao Dong

**Affiliations:** 1 Rail Transportation Technology Innovation Center, East China Jiaotong University, Nanchang 330013, China; 2 School of Electrical and Automation Engineering, East China Jiaotong University, Nanchang 330013, China; 3 Department of Mechanical and Electrical Engineering, Huazhong Agricultural University, Wuhan 430070, China

**Keywords:** wireless sensor network, soft multi-functional sensors, structural health monitoring, multi-agent system, damage detection

## Abstract

With the rapid growth of railways in China, the focus has changed to the maintenance of large-scale rail structures. Multi-agent systems (MASs) based on wireless sensor network (WSNs) with soft multi-functional sensors (SMFS) are adopted cooperatively for the structural health monitoring of large-scale rail structures. An MAS framework with three layers, namely the sensing data acquisition layer, sensor data processing layer, and application layer, is built here for collaborative data collection and processing for a rail structure. WSN nodes with strain, temperature, and piezoelectric sensor units are developed for the continuous structural health monitoring of the rail structure. The feature data at different levels are extracted for the online monitoring of the rail structure. Experiments carried out at the Rail Transmit Base at East China Jiaotong University verify that the WSN nodes with SMFS are successfully assembled onto a 100-m-long track for damage detection. Based on the sensing data and feature data, a neural network data fusion agent (DFA) is applied to calculate the damage index value of the track for comprehensive decisions regarding rail damage. The use of WSNs with multi-functional sensors and intelligent algorithms is recommended for cooperative structural health monitoring in railways.

## 1. Introduction

The operating mileage of China’s high-speed railway has exceeded 42,000 km [1], and it has changed to the long-term operation and maintenance stage from the large-scale construction stage [2]. It is more and more critical to manage and maintain the large-scale high-speed track lines [3]. New online inspection technology for long-distance tracks should be studied to ensure its safety and reliability, which can be used to collect multi-parameter data and allow the rapid detection of track defects via advanced signal sensing and processing technologies.

With long-term operation and wheel–rail interactions, fatigue cracks will happen, which rapidly expand and lead to accidents without treatment [4,5]. Wireless sensor network nodes with different sensors are laid out onto the rail waist for the structural health monitoring of the rail structure [6]. Conventional sensors have been applied to the health monitoring of the tracks based on the wayside detection method [5,7,8,9]. The Eddy current method with non-contact has been used to detect the cracks in the rail surface to determine the size and location of defects [10]. Acoustic emission technology (AET) is used for rail structure detection through non-destructive testing methods using the emitted stress waves [11]. However, these sensors have some disadvantages and limitations, such as their difficult installation, heavy weight, and being easy to be affected by electromagnetic interference [6]. An ultrasonic Lamb guided wave sensor is installed on the surface of the rail waist for the active detection of inner defects of the rail structure [12,13]. Wireless sensor networks (WSNs) have been applied for distributed cooperative structural health monitoring with different sensors [14]. WSNs with flexible polyvinylidene difluoride (PVDF) strain sensors are designed for wheel rail force measurements in railroad networks with high stability [15]. The architecture of a WSN is built for damage detection for distributed stress monitoring and field tests in high-speed railway tracks [16]. The use of intelligent sensing and advanced signal processing technologies for online monitoring of rail structure will help to improve the intelligent operation and maintenance level of the rail structure [17]. WSNs with flexible sensors have been applied for the long term structural health monitoring of engineering structures [18,19]. Flexible piezoelectric sensors are designed for defect detection on the curved surfaces of wind turbine blades [20]. However, these methods have some limitations, which would not be suitable for large-scale structural health monitoring. A distributed monitoring framework of the rail structure should be built to improve the efficiency of the sensing data and the recognition accuracy of the rail defects.

The use of a multi-agent system (MAS) framework for the structural health monitoring of rail structures is effective to complete the monitoring tasks cooperatively via the cooperation of the sensor agents. The strain, temperature, and piezoelectric sensors are embedded into the WSN node to collect the strain and temperature data from the tracks during the wheel or rail rolling process. A piezoelectric Lamb guided wave is applied to actively detect the structural health data from the track. The experiments on the rail transmit base in East China Jiaotong University have validated that the framework based on a WSN with soft sensors is effective for crack detection of track surfaces. The main contributions of this paper are the following:

(1) A multi-agent framework based on a WSN is adopted for collaborative structural health monitoring of the track with three layers: a distributed sensor data acquisition layer, sensor data processing layer, and application layer.
(2) WSN nodes with SMFS are successfully assembled onto a 100-m-long track for structural health monitoring and crack detection of the track. Based on the sensor data from the SA and feature data from SPA, neural network DFAs are applied to fuse the feature data for the crack detection of rail surfaces.

The rest of the paper is arranged as follows. An online monitoring framework based on a WSN with SMFS is built in Section 2. Section 3 depicts the distributed data collection and collaboration process based on the SA, SPA, and DFA. Experiments for damage recognition of the tracks are validated for distributed monitoring applications in Section 4. Finally, the whole text is concluded in Section 5.

## 2. Online Monitoring Framework of Rail Structure

### 2.1. Problem Formulation

**Figure 1:**
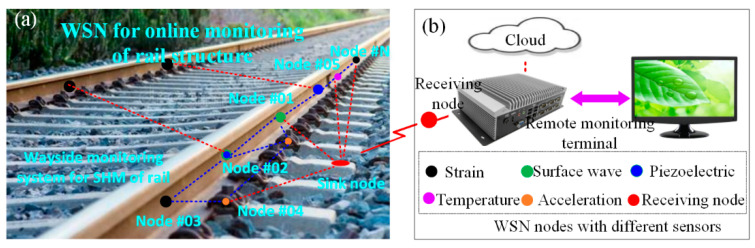
Long-distance rail structural health monitoring based on a WSN integrated with SMFS. (**a**) WSN nodes are laid out on the railway; (**b**) Monitoring terminal for long-distance rail.

For complicated environments and large-scale rail structures, new sensing devices and data processing methods are required for the structural health monitoring of the rail structure. WSN nodes with different sensors are arranged onto the rail to collect the multi-source signals of the rail structure, as shown in Figure 1, which are applied for the online monitoring of the rail structure. Soft strain, temperature, and acoustic surface wave sensors are integrated into the WSN nodes to collect the healthy data, which are applied to extract the feature data for the rail structure at different levels for online damage monitoring.

In large-scale rail structures, WSNs with SMFS are developed for the structural health monitoring of the rail structure. Due to the high-speed wheel–rail rolling, it would be more complicated to perform damage detection on the tracks. The cooperation mechanism for different WSN nodes is studied to ensure the consistency and coherence of the cooperation. Intelligent algorithms are proposed to optimize the information process for rail healthy data monitoring.

### 2.2. Collaborative Monitoring Framework for Rail Damage

**Figure 2:**
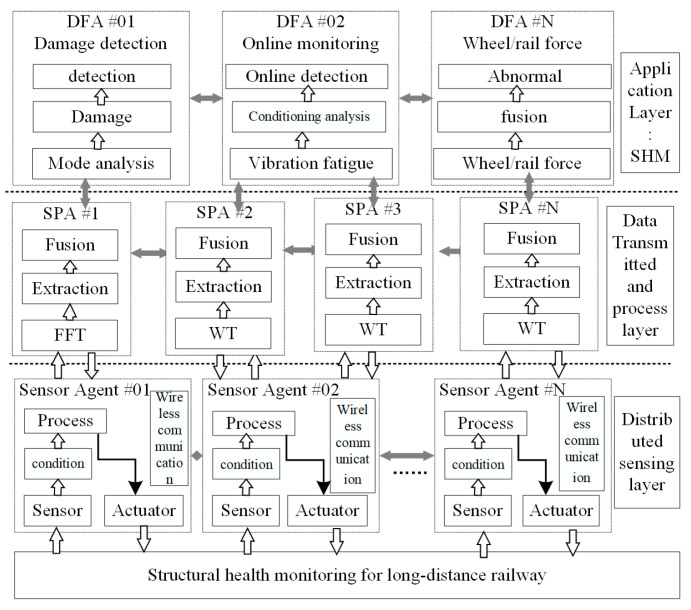
The monitoring architecture of large-scale rail structure based on MAS.

A WSN with SMFS is applied here for the structural health monitoring of a large-scale rail structure using distributed sensing data and an intelligent algorithm. An MAS based on the WSN is adopted for the distributed monitoring of the infrastructure [21], and a sensor model with smart agent technology is built to represent the sensor units for data collection. A data sharing mechanism is used for the cooperative sensing and damage detection of the monitored subjects. This provides an effective method of structural data collection and damage detection of the rail structure. The architecture based on the MAS for rail structure monitoring is shown in Figure 2, including three layers: the data sensing layer, data processing layer, and application layer. The sensor data are imported into the MAS architecture via distributed sensors in the data sensing layer. Different sensor agents are applied to collect the sensing data cooperatively, which are transmitted to the sensor processing agent (SPA) for feature extraction. The data fusion agents (DFA) are designed for damage identification of the rail structure.

(1) Data sensing layer

The data sensing layer is composed of various sensors laminated onto the surface of the rail structure to collect strain, vibration, pressure, and temperature data for structural health monitoring applications. The sensing data can be exchanged between different sensor agents to improve the data processing efficiency for the defect detection of long-distance rail structures.

(2) Data transmission and processing layer

In the data transmission and processing layer, different signal processing methods (such as FFT, HHT, wavelet analysis) are adopted for the feature extraction of the rail structure. An information processing and transmission module should be designed to process the multi-source sensing data from the data sensing layer. The SPA is designed for feature extraction from the sensing data, and the feature data are transmitted to the next layer for the damage detection of the rail structure.

(3) Damage identification layer

In the application layer used for damage identification, the neural network, genetic algorithm, and support vector machine are adopted to evaluate the feature data from the data transmission and processing layer, which are used for damage recognition under various operation conditions.

### 2.3. Principle of Agents

#### 2.3.1. Sensor Agent

**Figure 3:**
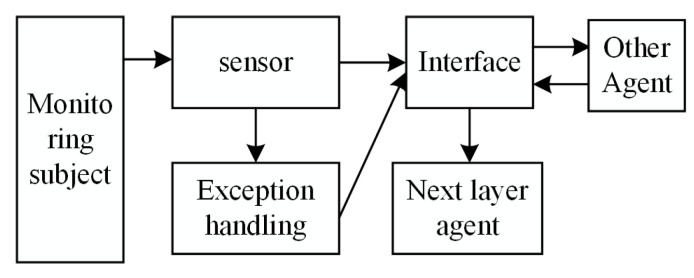
A structural diagram of an SA.

Sensor agents (SAs) are used to collect the structural health data from the different parts of the rail cooperatively. According to the condition–action mapping rules, they cooperates with other agents in collecting the structural health data. Figure 3 depicts a structural diagram of an SA for data processing, defined by ID, FD, and m, in which ID represents the unique identifier of the sensor agent. Different SAs are designed for structural health monitoring of tracks, including a piezoelectric sensing body (piezo.SA), temperature sensing body (temp.SA), and strain sensing body (strainSA). The processing results are sent to the data transmission and processing layer for feature extraction.

#### 2.3.2. Sensor Processing Agent (SPA)

**Figure 4:**
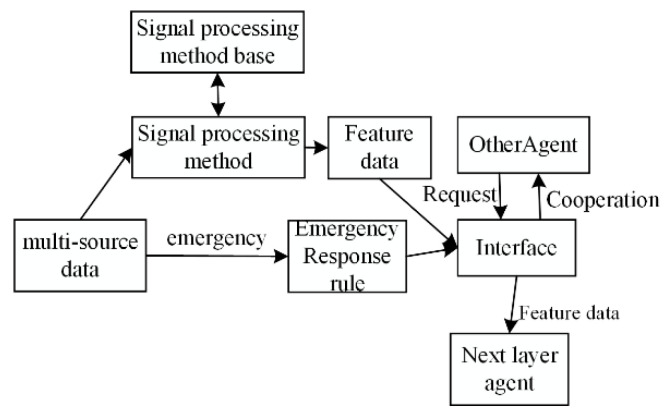
The internal structure and principle of the SPA.

The sensor processing agent (SPA) processes the structural rail health information from the sensor agent, and different data processing methods (e.g., fast Fourier transform (FFT), wavelet analysis agent, Hilbert transform) are integrated into the SPA to extract the feature data. The internal structure of the SPA is shown in Figure 4, and the transmission and processing agent is defined by ID, SD, and M, where ID is the unique ID number for the SPA. SD is the status information from the rail structure data from the SA, while M is the method of feature extraction. The damage features are transmitted to the next layer of the rail structure for damage recognition.

#### 2.3.3. Data Fusion Agent (DFA)

**Figure 5:**
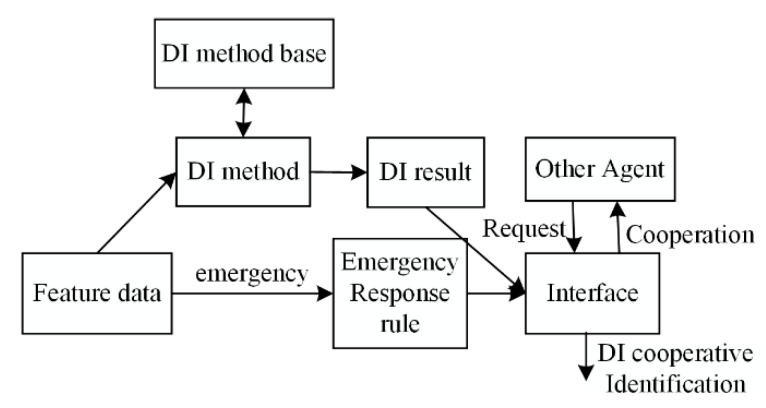
The internal structure and principle of the DFA.

The data fusion agent (DFA) is designed to fuse the feature data from different SPAs for damage recognition. The internal structural principle of the DFA is shown in Figure 5. The feature data from the SPA are used to evaluate the damage to the rail structure. DFAs are designed for structural damage assessments, mainly including statistical pattern recognition DFAs and neural network DFAs. Each DFA is defined by ID, FD, FM, and DR, where ID is the ID number of the DFA. FD is the state information of the rail structure, M is the feature fusion strategy, and DR is the damage fusion recognition result.

**Figure 6:**
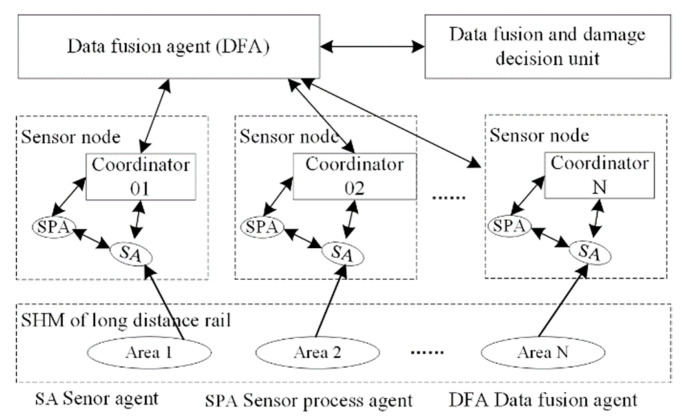
Online monitoring and damage identification of rail defect data by the MAS.

The online monitoring of the rail structure is decomposed into subtasks by different agents (SA, SPA, and DFA) in different layers in the proposed framework (Figure 2). A multi-agent cooperation mechanism is adopted to achieve the online monitoring and damage identification of the rail structure, as shown in Figure 6. A distributed cooperation framework based on the MAS is built to decompose the monitoring tasks of the large-scale rail structure into smaller areas. Data collection, processing, and fusion methods are adopted to process the multi-sensing data from the SMFS at different levels with different agents.

The large-scale rail structure is divided into several monitoring areas 1, 2, …, N, and WSN nodes with different sensors are arranged in the designated locations. The SA collects the structure information in the subareas, while the SPA extracts the feature data. Data communication and cooperation are implemented for the structural health monitoring of rails between the SPAs. The DFA is applied to fuse the feature data using different data fusion algorithms for damage identification. The cooperative data processing strategy between the sensor network nodes is adopted for the structural health monitoring of the rail structure, which is used for the cooperative damage identification of the rail structure.

## 3. Distributed Data Collection and Collaboration Process

### 3.1. Distributed Data Collection

#### 3.1.1. Active Damage Detection Based on Lamb Wave

**Figure 7:**
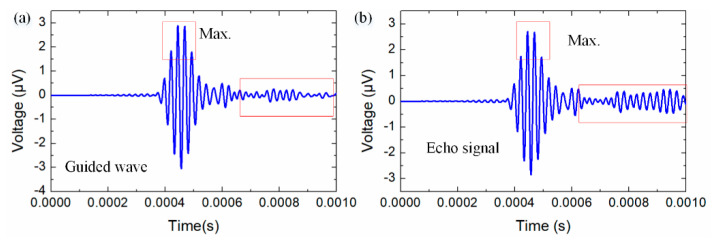
(**a**) Lamb guided wave and (**b**) echo signal for active damage detection of the rail.

The piezo.SA generates the Lamb guided wave signal, which spreads in the rail structure, and the echo signal is collected by the piezoelectric sensor, as shown in Figure 7. The feature data from the guided wave and echo signal are extracted for damage detection and recognition in the rail structure.

#### 3.1.2. Strain Sensor

**Figure 8:**
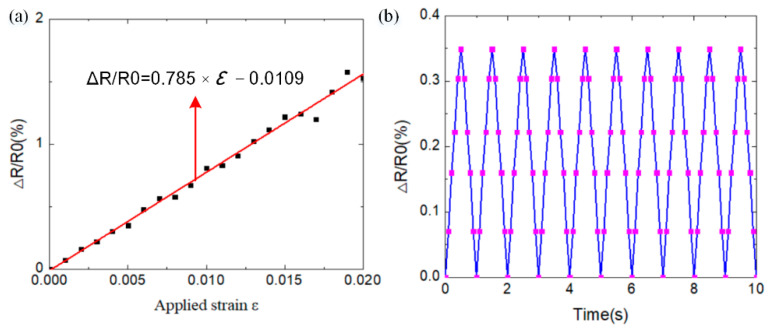
Strain sensor data from the strain sensor agent: (**a**) variable resistance value with the external strain; (**b**) repeated load and unload process for the strain sensor.

A strain sensor agent is integrated into the WSN nodes to collect the strain data from the rail structure under high-speed wheel–rail rolling. The strain sensor is designed with a flexible metal strain gauge to detect the external strain data. Figure 8 depicts the electrical performance of the strain sensor. It is shown that the variable resistance value of the strain sensor has a linear relationship with the external strain, as shown in Figure 8a. Figure 8b shows the good repetition properties of the strain sensor.

#### 3.1.3. Temperature Sensors

**Figure 9:**
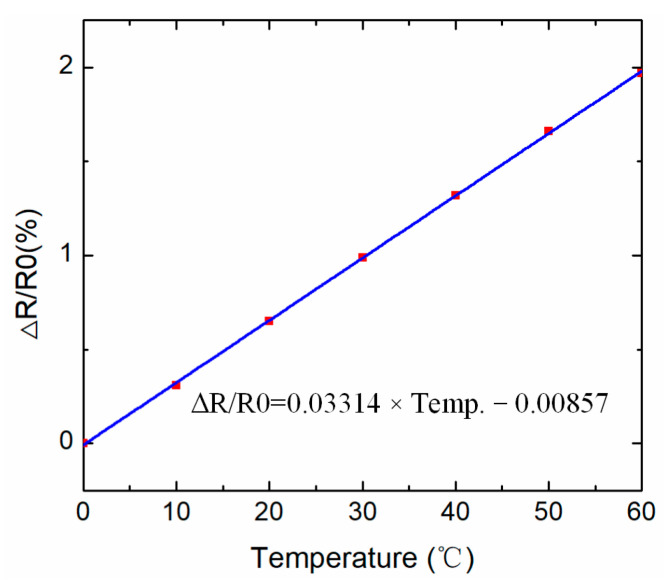
Temperature sensor data from the temperature sensor agent.

Temperature sensors with temperature-sensitive resistance are integrated into the WSN nodes to collect the temperature data for the rail structure in dynamic environments. Figure 9 shows the electrical performance of the temperature sensor, in which the variable resistance value of temperature sensor has a linear relationship with the variable temperature.

### 3.2. Feature Extraction

#### 3.2.1. Echo Signal from Lamb Wave

In the time domain, frequency domain and time–frequency domain, several physical parameters are selected to extract the feature data from the echo signal, such as the amplitude, root mean square (RMS), correlation coefficient, and spectrum loss. The amplitude, energy, and correlation coefficient are selected to evaluate the degree of rail damage. The amplitude is the peak value of the echo signal. The other feature data are illustrated as:

(1) $RMS_{e}$ is the RMS of the energy of the echo signal, which is represented as:

(1) $$RMS_{e}=\sqrt{\frac{1}{n}\sum_{i=1}^{n}e_{ei}^{2}},$$

where $e_{ei}^{}$ represents the time series data of the echo signal recorded by the piezoelectric sensor.

(2) The correlation coefficient $Cov\left(R,P\right)$ represents the correlation coefficient between the echo signal in the damage state and the signal without structural damage, which is represented by the correlation between the reference signal and the measured signal:

(2) $$\rho =\frac{Cov\left(R,P\right)}{\sigma_{R}\sigma_{P}},$$

where $Cov\left(R,P\right)$ is the covariance coefficient between the reference signal and the echo signal. Here, $\sigma_{R}$ and $\sigma_{P}$ are the standard deviations of reference signal and echo signal, respectively.

(3) The spectrum loss is an important index used to depict the performance of the echo signal in the frequency domain, which is represented as:

(3) $$SL=\frac{\int_{\omega_{1}}^{\varpi_{N}}\left|B\left(\omega \right)-D(\omega )\right|d\omega }{\int_{\omega_{1}}^{\varpi_{N}}\left|B\left(\omega \right)\right|d\omega },$$

(4) The central spectrum loss is represented as:

(4) $$CSL=\frac{a\left(\omega \right)-b\left(\omega \right)}{a\left(\omega \right)},$$

where $a\left(\omega \right)=\max\left(B\left(\omega \right)\right)$, $b\left(\omega \right)=\max\left(D\left(\omega \right)\right)$.

#### 3.2.2. Strain and Temperature Sensor Data

The feature data (amplitude, root mean square (RMS)) of the rail structure from the strain and temperature sensors are extracted for the structural health monitoring of the rail structure. The feature data are illustrated as:

(5) $RMS_{s}$ is the RMS of the strain sensing data:

(5) $$RMS_{s}=\sqrt{\frac{1}{n}\sum_{i=1}^{n}e_{si}^{2}},$$

where $e_{si}^{}$ represents the strain data from the strain data recorded by strain sensor.

(6) $RMS_{t}$ represents the RMS of the temperature sensing data:

(6) $$RMS_{t}=\sqrt{\frac{1}{n}\sum_{i=1}^{n}e_{ti}^{2}},$$

where $e_{ti}^{}$ represents the temperature data for the echo signal recorded by the temperature sensor.

### 3.3. Feature Fusion

**Figure 10:**
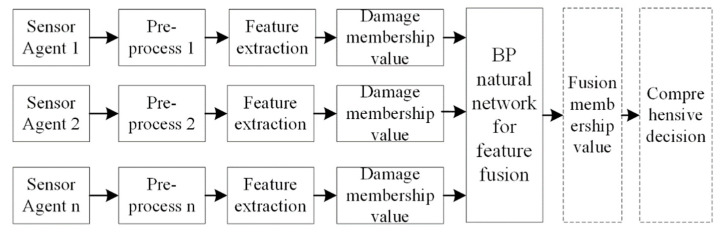
Data fusion framework for track damage detection.

Multi-sensing data from the rail structure are obtained from the SA in dynamic environments for wheel–rail interactions. The WSN with SMFS is designed for strain, temperature, and echo signal data collection. SPAs are applied to extract the feature data (Equations (1)–(6)) for the strain, temperature, and echo signal. DFAs are applied to fuse the features from the SPAs for damage recognition. Figure 10 depicts the data fusion algorithm for damage recognition based on the multi-source data, for which the SAs, SPAs, and DFAs process the sensing data cooperatively. Piezoelectric, temperature, and strain sensors embedded into the WSN are applied to collect the structural health data at the sensor agent level. The SPA with different signal processing methods is adopted to extract the feature data for the rail structure (dynamic strain, surface acoustic wave, and temperature). An empirical mode method is adopted to process the acoustic surface wave signal, and an FFT is used to extract feature data for the wheel rail vibration signal. A neural network is adopted for multi-source feature data fusion under the wheel rail rolling area [22]. The feature data are imported into the natural network to compute the damage index (DI) value, which reflects the health state of the track. DI is the function of the feature data (Equations (1)–(6)), which can be represented as ***DI = DI (RMS_e_, ρ, SL, CSL, RMS_s_, RMS_t_*)**. The DI value gives important evidence for a comprehensive evaluation of the track damage.

## 4. Experiment for Track Damage Recognition

### 4.1. Experimental Setup

**Figure 11:**
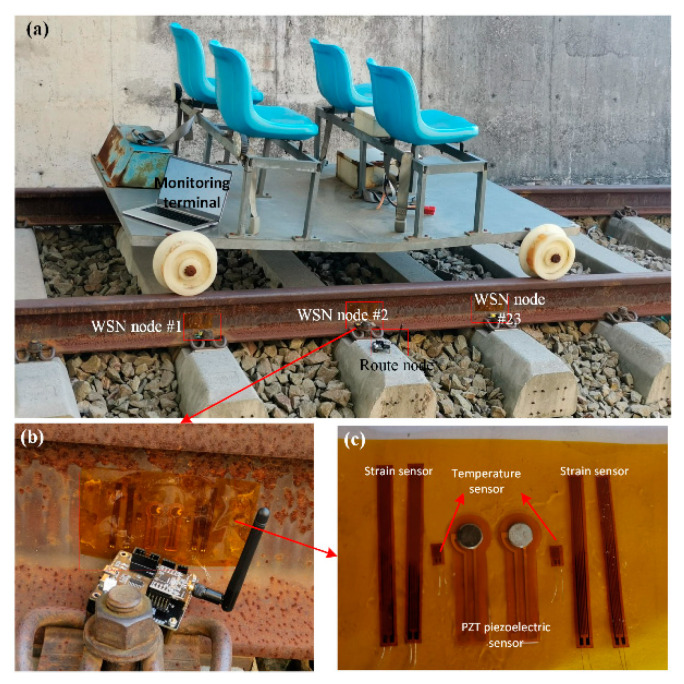
(**a**) Experimental setup for the online structural health monitoring of rail structures based on an MAS with SMFS; (**b**) optical image of WSN with SMFS; (**c**) optical image of SMFS.

The WSN nodes with SMFS were arranged on the rail structure (~100 m distance) at the Rail Transit Base of East China Jiaotong University, as shown in Figure 11. With the collaborative monitoring framework (Figure 2), the cooperation mechanism of the MAS based on WSN is proposed for online structural health monitoring for the damage detection and identification of rail structures (Figure 11a). WSN nodes integrated with piezoelectric, strain, and temperature sensors are arranged onto the rail structure (rail waist, fasteners), as shown in Figure 11b, which are applied for collaborative structural health monitoring of the long-distance rail. An enlarged image of the soft sensors is shown in Figure 11c, showing the piezoelectric guided wave sensor, two temperature sensors, and four strain sensors. Sensor agents (piezoelectric guided wave sensor agent, temperature sensor agent, and strain agent) are adopted to collect the health data from the rail structure, which are used as the input data for the SPA for feature extraction. A data fusion agent (DFA) is used for damage recognition of the rail structure through the multi-source feature data fusion algorithm. This provides the guidelines for the experiment to validate the cooperative mechanism of the data processing based on the MAS.

### 4.2. Online Structural Health Monitoring of Rail Structures

**Figure 12:**
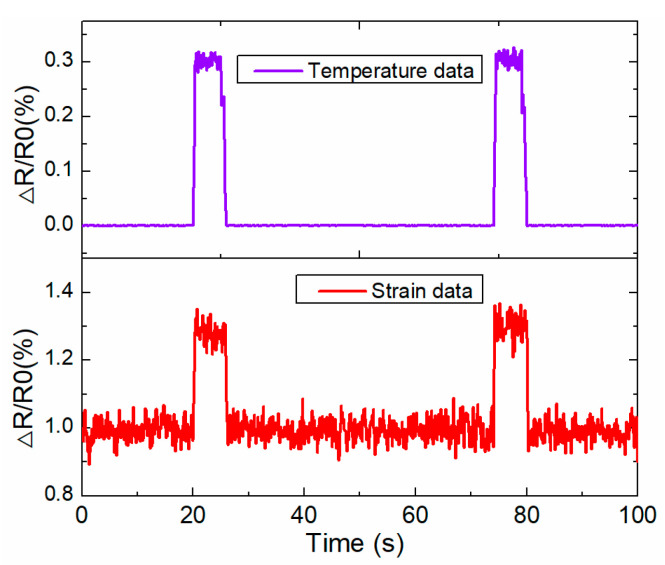
The strain and temperature sensing data from the SMFS embedded into WSN nodes for cooperative data collection from the track.

A cooperative sensing and signal processing strategy is studied here for the structural health monitoring of a rail structure based on a WSN embedded with SMFS. The strain, temperature signal, and acoustic surface wave information are collected by the different sensor agents embedded into WSN, which is applied for damage recognition for the rail structure. The sensing data are transmitted to the SPA layer in the built framework based on the MAS for further data processing. The strain and temperature sensing data are collected by the strain and temperature sensor agents, as shown in Figure 12. Peaks in the temperature and strain values occur when the track inspection car runs onto the track (WSN nodes are installed on this part). In [23], it was demonstrated that the resistance value of a soft strain sensor increase with an increasing crack size. The peak value of the strain sensor would be larger if the crack length on the track was bigger, which is helpful for damage detection based on the output of the strain sensor. At about 20 s, Figure 12 depicts the temperature and strain sensor data increases as the track inspection car runs on the rail, which would induce deformation of the track due to the wheel–rail rolling interaction. If the track inspection car does not run on the track, the temperature and strain sensor data would be kept stable as the environment of the rail would not change during this time.

**Figure 13:**
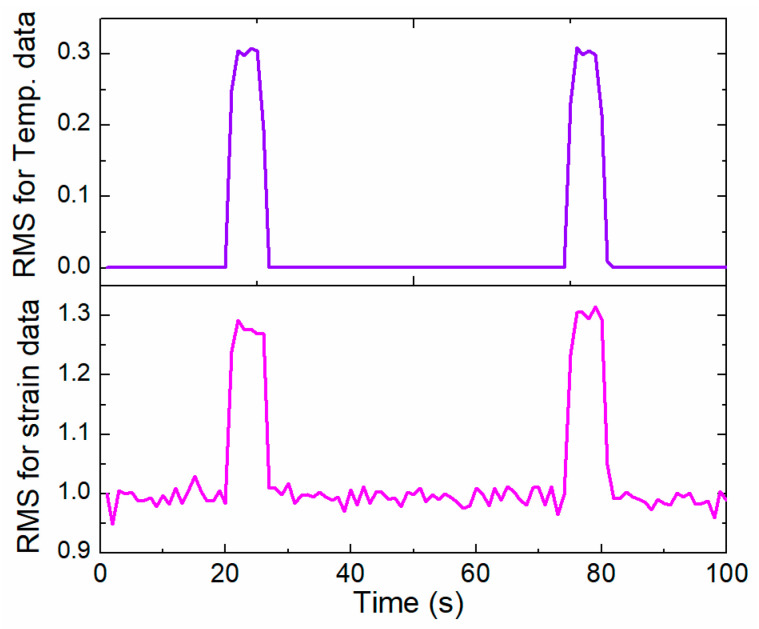
RMS value for the strain and temperature data extracted by the SPA.

For the temperature and strain sensing data from the sensor agents, the feature signals (RMS) are extracted to evaluate the healthy information of the track by SPA. Figure 13 depicts the feature signals extracted from the sensing data (Figure 12). It is shown that the RMS of the temperature and strain data has a similar trend to the original sensing data. The feature data would be applied to replace the sensing data and transmitted to the DFA for damage recognition for the track.

**Figure 14:**
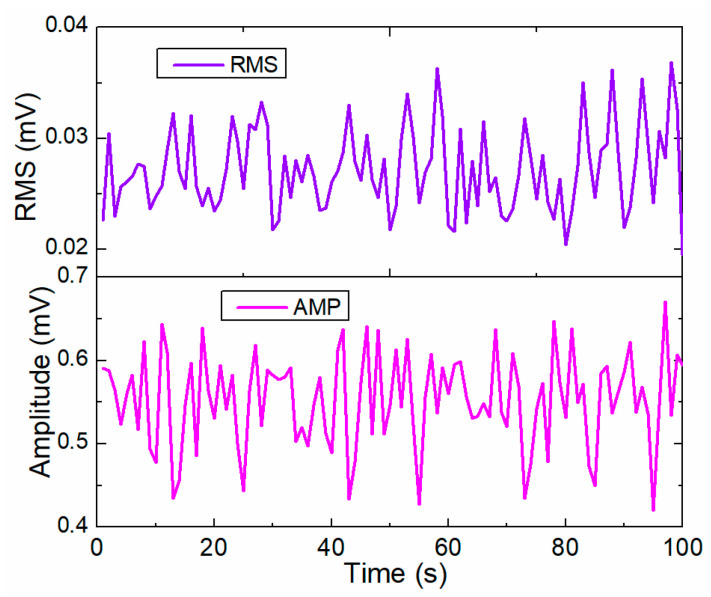
RMS and aptitude information from the echo signal processed by the SPA.

The guided Lamb wave is generated by the piezoelectric PZT actuator and the echo signal is received by the PZT sensor unit, as shown in Figure 7. The piezoelectric guided wave sensor agent generates the Lamb wave signal with a sine five peak wave (center frequency: 10 kHz) under the external signal generator. The Lamb wave is applied to actively detect the damage information from the rail structure and the echo signal is received by the piezoelectric sensor. Comparing the echo signal to the Lamb wave signal, the feature information (Equations (1)–(4)) is extracted for damage detection in the rail structure. The RMS, correlation coefficient, and spectrum loss are selected as the feature data for the echo signal to illustrate the health data for the rail structure. Figure 14 depicts the RMS and aptitude feature data for the echo signal processed by the SPA. The feature data are transferred to the DFAs for a compressive analysis of the rail structure.

**Figure 15:**
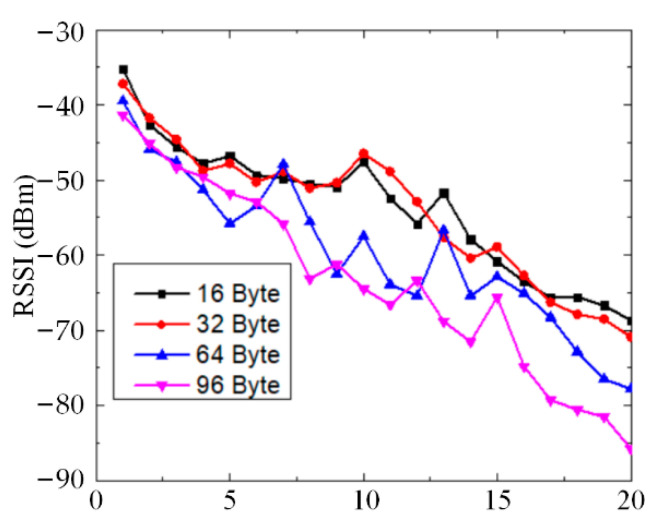
RSSI values from WSN communication with different payloads.

WSNs embedded with SMFS have been applied for the structural health monitoring of long tracks, and their wireless communications ability is illustrated by their RSSI values, as shown in Figure 15. The RSSI value decreases with the communication distance with different payloads, whereby the RSSI value is large enough to ensure the wireless data communication ability. Figure 15 shows that the WSN nodes could transmit 16 bytes, 32 bytes, 64 bytes, and 96 bytes to the other nodes. The data transfer rate of the WSN is 9600 bps using the wireless ZigBee communication protocol. It is indicated that the data transfer capacity reaches 96 bytes for data sharing of the health state of the track.

**Figure 16:**
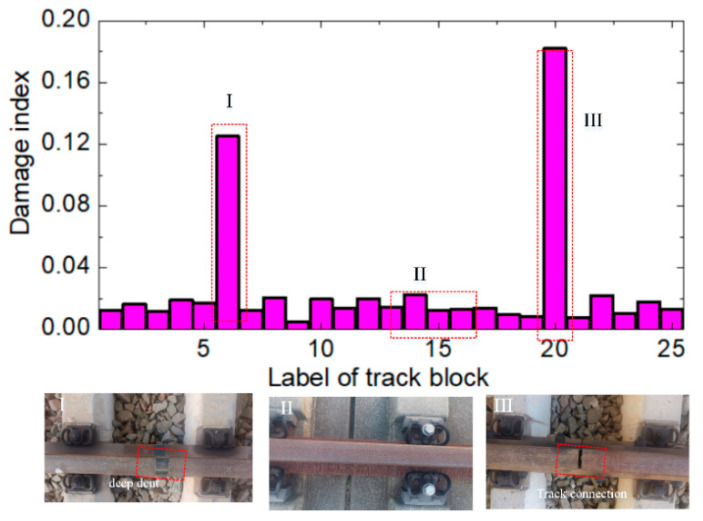
Damage index values of the rail structure based on WSNs embedded with SMFS.

WSNs with SMFS were assembled onto the 100-m-long track, and the 100-m-long track was divided into 25 blocks (4 m is one block), whereby one WSN node is placed onto each block. Figure 16 depicts the computation results (DI values of around 0.03 to 0.18) for the 100 m track with the 25 blocks. Based on the sensing data from the SA and feature data (Equations (1)–(6)) from the SPA, neural network DFAs are adopted to calculate the damage index (DI) value of the track with the multi-sensing data. The DI value is represented as ***DI = DI (RMSe, ρ, SL, CSL, RMSs, RMSt*)**. It is shown that the DI values for the 25 blocks are around 0.03 to 0.18, for which labels 6 and 20 of the block are larger than the others. The experiments show that label 6 of the track block is damaged and the surface of the track has a deep dent, as shown in the optical image in Figure 16 (I). Label 20 of the track block is the connection point of the two tracks, and there is a big gap, as shown in the optical image in Figure 16 (III). The abnormal signal of the track block is collected by the WSN with soft sensors, which agree well with practical case. This would lead to high-frequency vibrations and high shock forces when the track inspection car runs onto the track. The abnormal signal of the wheel–rail interaction was collected using the WSN. The DI values of the other blocks of track are smaller due to the wheel–rail rolling and environment effects, which show that these blocks are safe without defects. WSNs with SMFS would be effective for the online structural health monitoring of tracks, which would be helpful for the damage location identification of tracks based on an MAS.

## 5. Discussion and Conclusions

A multi-agent framework based on WSNs with soft multi-functional sensors (SMFS) was built for the collaborative data sensing and damage detection of a rail structure, which included three layers: the sensor data acquisition layer, sensor data processing layer, and application layer. WSN nodes with strain, temperature, and piezoelectric sensing units were developed for the continuous structural health monitoring and damage detection of the rail structure. The main findings of our work are concluded as follows:

(1) A multi-agent framework was built for the structural health monitoring of a rail structure based on a WSN with three layers: the sensor data acquisition layer, sensor data processing layer, and application layer. The distributed data sensing method was used to collect the sensing data from the different blocks in the long-distance rail structure by the SAs, and feature data at different levels were extracted by the SPA for online monitoring of the rail structure.
(2) The experiments verified that the WSN with SMFS was successfully assembled onto the 100-m-long track for crack detection of the rail surface. Based on the sensing data from the SA and feature data from the SPA, the DFAs were designed to calculate the damage index (DI) of the track with the multi-sensing data through an intelligent fusion algorithm for damage recognition.

WSNs with SMFS have been applied for the continuous structural health monitoring of tracks for damage detection. However, the performance of WSNs with SMFS could be improved in several aspects:

(1) WSN nodes with more functionality should be developed for the structural health monitoring of tracks. A flexible hybrid electronic technology could be adopted for WSN nodes to improve the integration ability of sensing devices with good electrical and communication performances;
(2) Field tests for rail monitoring could be implemented using WSNs with soft sensors. This would be useful to improve the electrical performance of WSNs with soft sensors during long-term structural health monitoring in complicated environments due to the high-speed wheel–rail rolling interactions.

## Data Availability

All data will be provided upon reasonable request to the corresponding author.
